# Patterning of the *Drosophila* retina by the morphogenetic furrow

**DOI:** 10.3389/fcell.2023.1151348

**Published:** 2023-04-06

**Authors:** Jasmine Warren, Justin P. Kumar

**Affiliations:** Department of Biology, Indiana University, Bloomington, IN, United States

**Keywords:** *Drosophila*, eye, pattern formation, morphogenetic furrow, morphogen, diffusion-reaction, positional information, cell flow

## Abstract

Pattern formation is the process by which cells within a homogeneous epithelial sheet acquire distinctive fates depending upon their relative spatial position to each other. Several proposals, starting with Alan Turing’s diffusion-reaction model, have been put forth over the last 70 years to describe how periodic patterns like those of vertebrate somites and skin hairs, mammalian molars, fish scales, and avian feather buds emerge during development. One of the best experimental systems for testing said models and identifying the gene regulatory networks that control pattern formation is the compound eye of the fruit fly, *Drosophila melanogaster*. Its cellular morphogenesis has been extensively studied for more than a century and hundreds of mutants that affect its development have been isolated. In this review we will focus on the morphogenetic furrow, a wave of differentiation that takes an initially homogeneous sheet of cells and converts it into an ordered array of unit eyes or ommatidia. Since the discovery of the furrow in 1976, positive and negative acting morphogens have been thought to be solely responsible for propagating the movement of the furrow across a motionless field of cells. However, a recent study has challenged this model and instead proposed that mechanical driven cell flow also contributes to retinal pattern formation. We will discuss both models and their impact on patterning.

## Introduction

**FIGURE 1:**
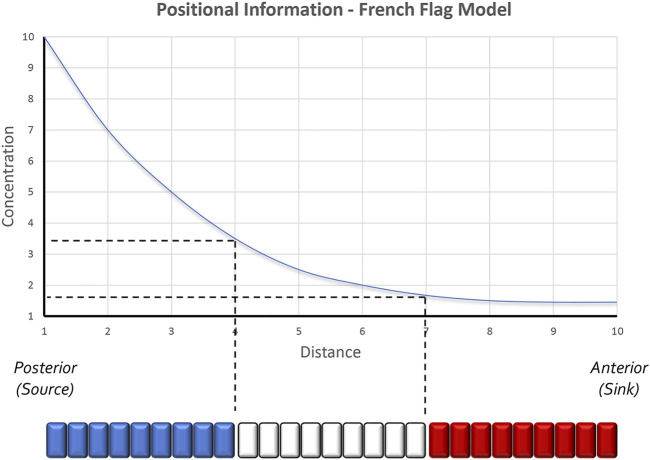
The positional information model of pattern formation. Based in part by the results of transplantation experiments conducted within the chick limb bud by John Saunders, Lewis Wolpert proposed that secreted morphogens form smooth gradients across developing tissues. Groups of cells along the gradient then capture unique amounts of the morphogen and as a result produce distinct structures. Colorized representations of this model are often represented as a French Flag where each color of the flag represents the conversion of a distinct morphogen concentration into a unique physical structure. Studies in the *Drosophila* embryo have further suggested that even 2 cells lying adjacent to each other can sense very small differences in morphogen concentrations and as a result execute different developmental programs. The schematic is adapted from Sharpe and Greene, 2015.

Once the fate of an initially homogeneous tissue has been specified, each cell must adopt a specialized fate—This is the process of pattern formation, a term coined by the great developmental biologist Lewis Wolpert. To explain how tissue patterning occurred, Wolpert formulated the concept of positional information (Figure 1). At its core, his model predicts that every cell can sense its relative location within an epithelium and adopt a fate that is appropriate for its position within the developing field (Wolpert, 1969). His thinking was influenced in part by the work of John Saunders who had demonstrated that the posterior margin of the chick wing bud, when transplanted to the anterior margin, would force the anterior domain into producing digits that were normally associated with the posterior half of the limb bud. As a result, a mirror-symmetry duplication of digits was generated across the anterior-posterior axis (Saunders and Gasseling, 1968). Wolpert proposed that the posterior region of the limb bud produced a long-range morphogen that established a concentration gradient across the entire posterior-anterior axis (Wolpert, 1969). He suggested that cells lying along this axis could sense and interpret negligible differences in morphogen titer or exposure time and this in turn would result in the specification of distinct cell fates (in this case, distinct digits). Support for Wolpert’s ideas of patterning came from the discovery in both chick and mouse that Sonic hedgehog (Shh) is expressed within the posterior limb bud, that its loss leads to the elimination of posterior digits, and that the induction of digit duplications that were seen in grafting experiments could be recapitulated if Shh expressing cells or beads soaked with Shh were transplanted into the anterior domain of the limb bud (Echelard et al., 1993; Riddle et al., 1993; Chang et al., 1994; Lopez-Martinez et al., 1995; Pagan et al., 1996; Yang et al., 1997). Additional support for Shh functioning as a morphogen came from experiments showing that the extent of digit duplications was directly proportional to the concentration of Shh (Yang et al., 1997). Manipulating exposure time demonstrated that the duration of Shh exposure is also important for limb patterning (Yang et al., 1997; Ahn and Joyner, 2004; Harfe et al., 2004).

**FIGURE 2:**
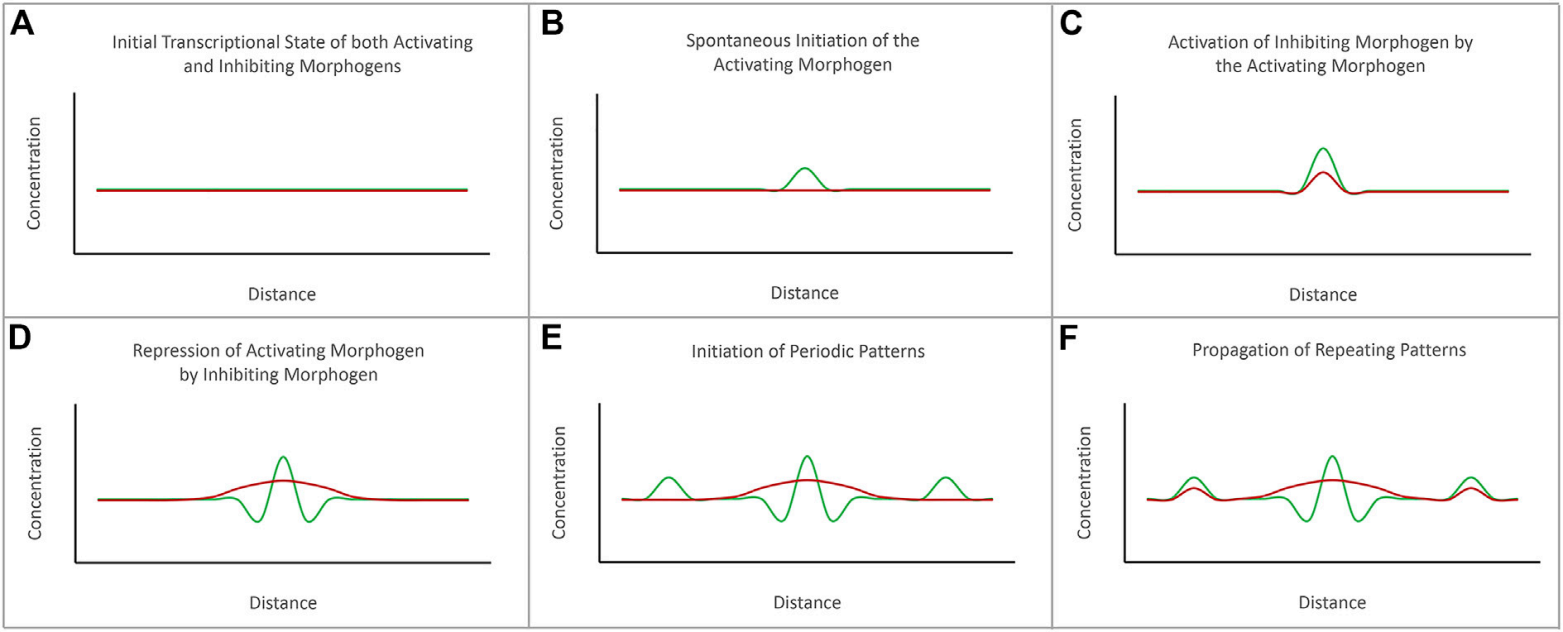
The diffusion-reaction model for the *de novo* initiation of repeated patterns. Alan Turing proposed that repeated patterns could spontaneously be generated *via* the combined activities of activating and inhibiting morphogens. **(A)** Prior to the initiation of pattern formation, the expression levels of both activating and inhibiting morphogens are at baseline levels. **(B)** The initiation of a pattern begins with the spontaneous initiation of expression of the activating morphogen. **(C)** As levels of the activating morphogen rises, it activates expression of the inhibiting morphogen. **(D)** The expression levels of the activating morphogen are higher than the inhibiting morphogen at the source. However, the inhibiting morphogen can diffuse further than the activating morphogen. **(E)** The inhibiting morphogen suppresses the expression of the activating morphogen further away from the source. However, just beyond the range of the inhibiting morphogen is seen spontaneous activation of the activating morphogens. **(F)** The process repeats itself to generate a periodically spaced pattern of repeated elements. The schematic is adapted from Sharpe and Green, 2015.

Although Wolpert is most associated with the concept of morphogen gradients, many researchers in preceding decades had proposed that concentration gradients of secreted molecules could be the genesis for spatial patterns (Boveri, 1901; Morgan, 1901; Child, 1915; Runnstrom, 1929; Dalcq and Pasteels, 1938; Horstadius, 1939). Their ideas were based on the observation that changes in spatial patterns after tissue transplantation or extirpation were quantitative and thus might be subject to changes in the concentration of a diffusible substance. The relationship between Shh concentration and digit duplications in the chick limb (i.e., increasing Shh titer = more complete duplication) is a classic confirmation of the concentration gradient hypothesis (Yang et al., 1997). Interestingly, a recent synthesis has suggested a “neighborhood watch” model (Figure 2) in which cells interpret positional information not by autonomously sensing their address within a morphogen gradient but rather by comparing themselves to their neighbors (Lee et al., 2022). This innovative advance to Wolpert’s original positional information model excellently explains how patterns can be generated across very large distances.

**FIGURE 3:**
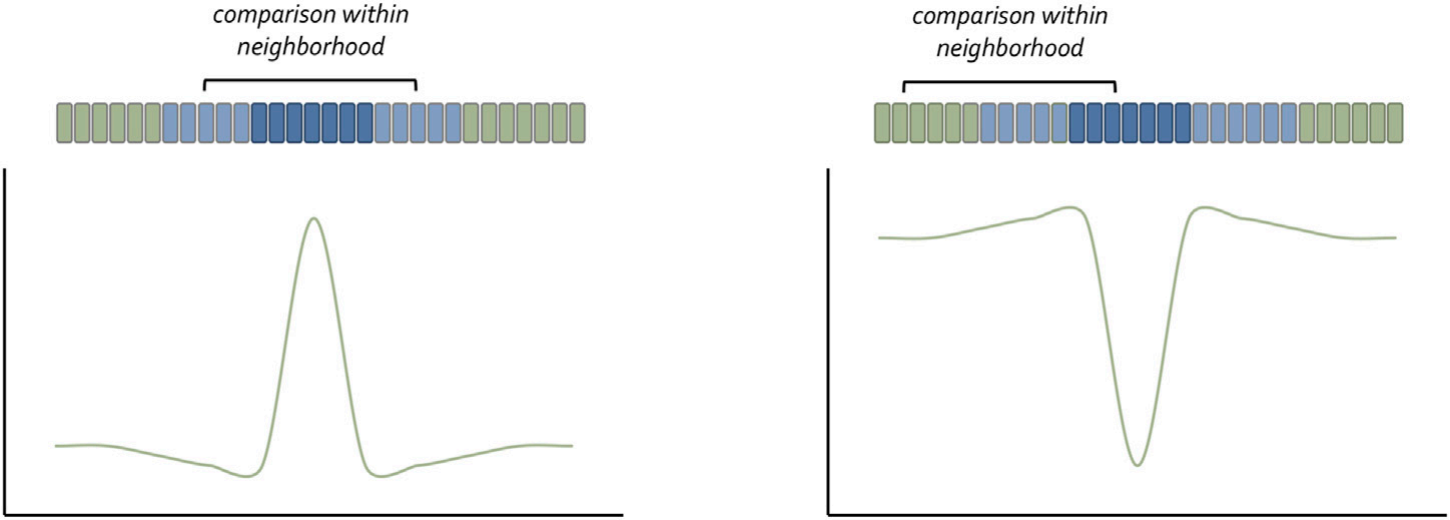
The neighborhood watch model for understanding how cells understand their position within a concentration gradient. Claudio Stern has recently proposed that a cell cannot interpret the absolute value of the morphogen it captures. Instead, it compares itself with its neighbors to make a relative calculation as to the amount of morphogen it has received. The schematic was adapted from Lee et al., 2022.

Alan Turing, the mathematician and World War II era codebreaker, was a notable contributor to the field of developmental biology through the publication of his diffusion-reaction model nearly two decades before Wolpert’s model (Turing, 1952). He coined the term “morphogen” and proposed that the production of two morphogens (one that serves as an activator and one that serves as a repressor) could generate spatial patterns within a *de novo* homogeneous field by antagonizing each other’s activity (Figure 3). But for the two signals to not simply cancel each other out, two criteria had to be met. First, the activator must be produced at a higher concentration than the repressor in order to overcome local repression. Second, the repressor needs to have long-range effects (diffuse fast and far) while the activator needs to be a short-range signal (diffuse slowly and over only a short distance). While he did not predict it, differences in the affinities for ligands and receptors can also play a role in limiting the reach of the activator. Turing’s model also predicted that the size and shape of the generated pattern would correspond directly to the distribution of the activator morphogen gradients (i.e., the width of a spot or stripe would correspond to the distance that the activator morphogen diffused).

The diffusion-reaction model as proposed by Turing stands in sharp contrast to Wolpert’s positional information model. Turing’s interest was in explaining how a spatial pattern could be generated *de novo* from a homogenous tissue, while Wolpert was attempting to understand how patterns developed in a field in which pre-existing molecular heterogeneities already existed. Another important difference is that the diffusion-reaction model predicts that the morphogen itself produced the observed spatial pattern while the positional information model requires that cells sense and interpret the amount of morphogen to which they were exposed. In practical terms, Turing’s proposal implied that the size and shape of the spatial pattern was directly correlated to the size and shape of the morphogen gradient. In contrast, Wolpert firmly believed that a smooth concentration gradient (high at the source to low at the edge of the sink) could generate any type of pattern. While these two models stand in apparent opposition to each other in the minds of many developmental biologists, a relatively recent synthesis has suggested potential avenues for how these two important ideas can be incorporated into a single model for pattern formation (Green and Sharpe, 2015).

A special circumstance in which the diffusion-reaction and positional information models have been particularly useful is the generation of simple repeated patterns. Some of the most studied examples include the scales of fishes (Bertin, 1944; Breder, 1947; Gunter, 1948), feather buds of birds (Holmes, 1935; Wessells, 1965), somites of vertebrates (Cooke, 1975; Cooke and Zeeman, 1976), molars and hair follicles of mammals (Gaunt, 1955; Straile, 1960; Mann, 1962; Lumsden, 1970; Mou et al., 2006; Prochazka et al., 2010; Cheng et al., 2014), and the unit eyes or ommatidia of the *Drosophila melanogaster* compound eye (Ready et al., 1976). The repeated nature of these systems means that a mutation which affects one element of the pattern affects the entire assembly. As such, mutations that disrupt repeated patterns have outsized effects that are easy to identify. Genetic screens and target gene knockdowns have shown that each of the above repeated patterns is disturbed by reductions in Shh or it orthologs (Heberlein et al., 1993; Ma et al., 1993; Fan and Tessier-Lavigne, 1994; Johnson et al., 1994; Hardcastle et al., 1998; St-Jacques et al., 1998; Chiang et al., 1999; Karlsson et al., 1999; Cobourne et al., 2004; Busby et al., 2020), which is entirely consistent with hypotheses put forth by Turing and Wolpert. Here, we will focus on how the morphogenetic furrow patterns the *Drosophila* eye. In this context, we will discuss the roles that several signaling pathways including Hedgehog (Hh), Decapentaplegic (Dpp), Wingless (Wg), Notch (N), and the EGF Receptor (EGFR) pathways play in regulating the initiation and progression of the morphogenetic furrow. We will also discuss how these signaling cascades generate interlocking columns of periodically spaced unit eyes.

The initial descriptions of the morphogenetic furrow by Donald Ready (Ready et al., 1976; Lebovitz and Ready, 1986; Wolff and Ready, 1991) and the subsequent identification of a role for the Hh morphogen in patterning by Kevin Moses and Ulrike Heberlein (Heberlein et al., 1993; Ma et al., 1993) suggested that diffusion-reaction and positional information models are likely sufficient to explain how the compound eye is patterned. Conspicuously absent from these discussions of the compound eye were models centered around mechanical forces contributing to the emergence of biological patterns. In general, mechanisms such as these were explicitly rejected by both Turing and Wolpert. However, a recent study from Richard Carthew has provided a compelling reason to consider the validity of mechanical forces such as cell flow in pattern formation. We will discuss the evidence supporting both chemical and mechanical force models for patterning.

## Structure of the adult *Drosophila* eye and the eye-antennal disc

**FIGURE 4:**
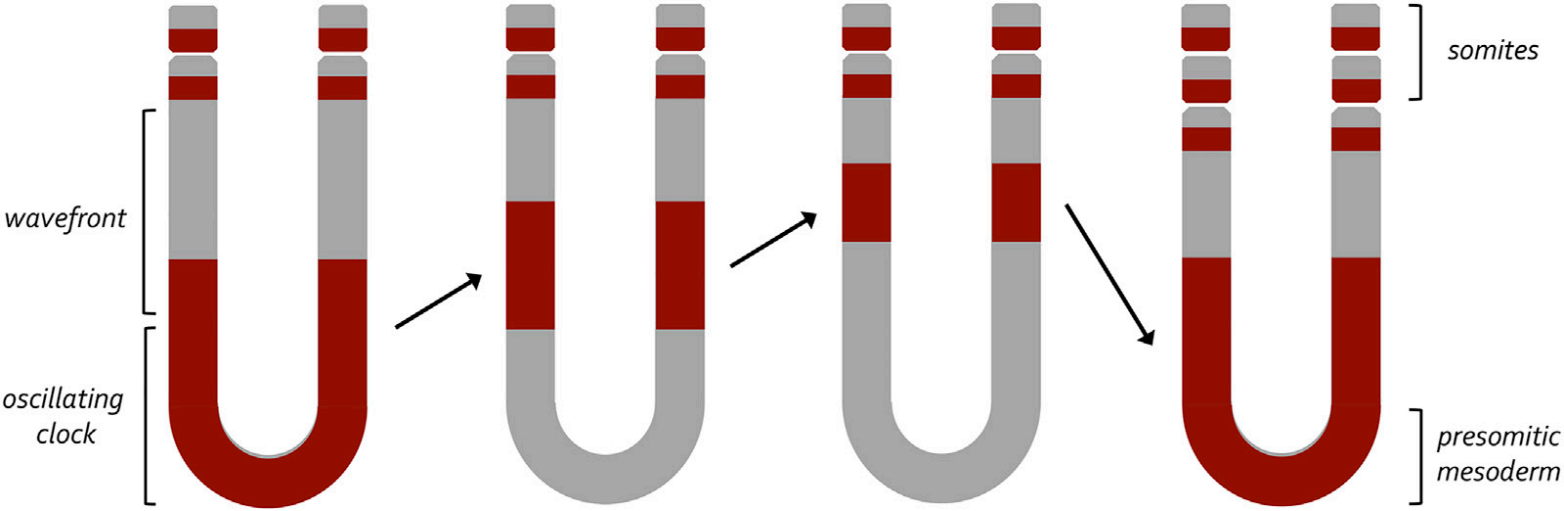
A clock and wavefront model for the generation of vertebrate somites. This model, developed by Johnathan Cooke and Erick Christopher Zeeman, proposed a two-component mechanism that would account for the periodic emergence of vertebrate somites. At its core it proposes that a wavefront of maturation (i.e., gene expression) transforms populations of undifferentiated cells into a pair of somites at periodic intervals. These periodic waves are triggered by an internal oscillator within the pre-somitic mesoderm (clock). Initially, it was thought that a similar internal oscillator could participate in the production of column of ommatidia within the fly eye. The variable rate at which these columns are now known to be produced suggests that an internal clock does not exist within the fly eye imaginal disc.

**FIGURE 5:**
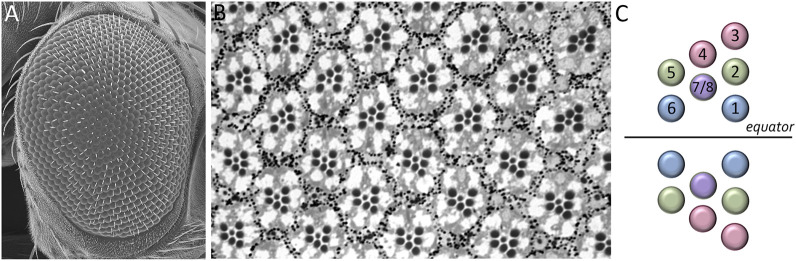
Structure of the adult *Drosophila* compound eye. **(A)** A scanning electron micrograph of the adult compound eye reveals that it consists of approximately 750 unit eyes or ommatidia that are organized into 32–34 columns. **(B)** A light microscope section of the adult retina shows that the eight photoreceptors that are contained within each unit eye are organized into an asymmetrical trapezoid pattern. The only difference between one unit eye and another is the chirality of the trapezoid within ommatidia. **(C)** A schematic showing the distinct chiral patterns of ommatidia within the dorsal and ventral compartments. These two compartments meet at the center of the eye which is called the equator.

The adult *Drosophila* eye is a simple nervous system comprised of approximately 750 ommatidia that are packed into a hexagonal array consisting of 32–34 interlocking vertical columns (Figure 4) (Ready et al., 1976). The compound eye is responsible for a wide range of visual and circadian behaviors (Grenacher, 1879; Exner, 1891; Mallock, 1894; Horridge, 1975; Strausfeld, 1976; Heisenberg and Buchner, 1977; Fischbach, 1979; Paulus, 1979; Land and Fernald, 1992; Vosshall and Young, 1995; Land, 1997; Veleri et al., 2007; Schlichting et al., 2016; Schnaitmann et al., 2020). These behaviors are augmented by the ocelli, which are three simple eyes located on the head vertex (Medioni, 1959; Fischbach and Reichert, 1978; Hu and Stark, 1980; Rieger et al., 2003; Umezaki and Tomioka, 2008; Krapp, 2009; Saint-Charles et al., 2016; Jean-Guillaume and Kumar, 2022). Each ommatidium within the compound eye contains eight photoreceptor neurons, four lens-secreting cone cells, a set of optically insulating pigment cells, and a mechanosensory bristle complex. Since each cell occupies a stereotyped position within the ommatidium, every unit eye is an exact replica of its neighbors (Waddington and Perry, 1960; Ready et al., 1976; Tomlinson and Ready, 1987; Cagan and Ready, 1989a). This is best seen in retinal sections of the adult retina with the only difference between ommatidia is that the photoreceptors within dorsal half of the eye are organized in a chiral pattern that is the mirror opposite to those within the ventral half of the eye. The two chiral versions of ommatidia meet at the equator, which is an invisible line that runs along the middle of the compound eye (Figure 4). Mutations that affect the development of individual cells within the ommatidium affect the overall structure of the unit eye. In rare instances, a cell within a single ommatidium of a genetically normal fly will fail to develop correctly and this will manifest itself as a small, barely visible, local distortion in the array. In contrast, genetic mutations that affect the specification of one or more cells in every ommatidium can be identified by a “roughening” or “glazing” of the entire external surface and/or a reduction in the overall size of the compound eye. In some instances, the compound eye is completely lost and replaced with epidermal tissue (Figure 5).

**FIGURE 6:**
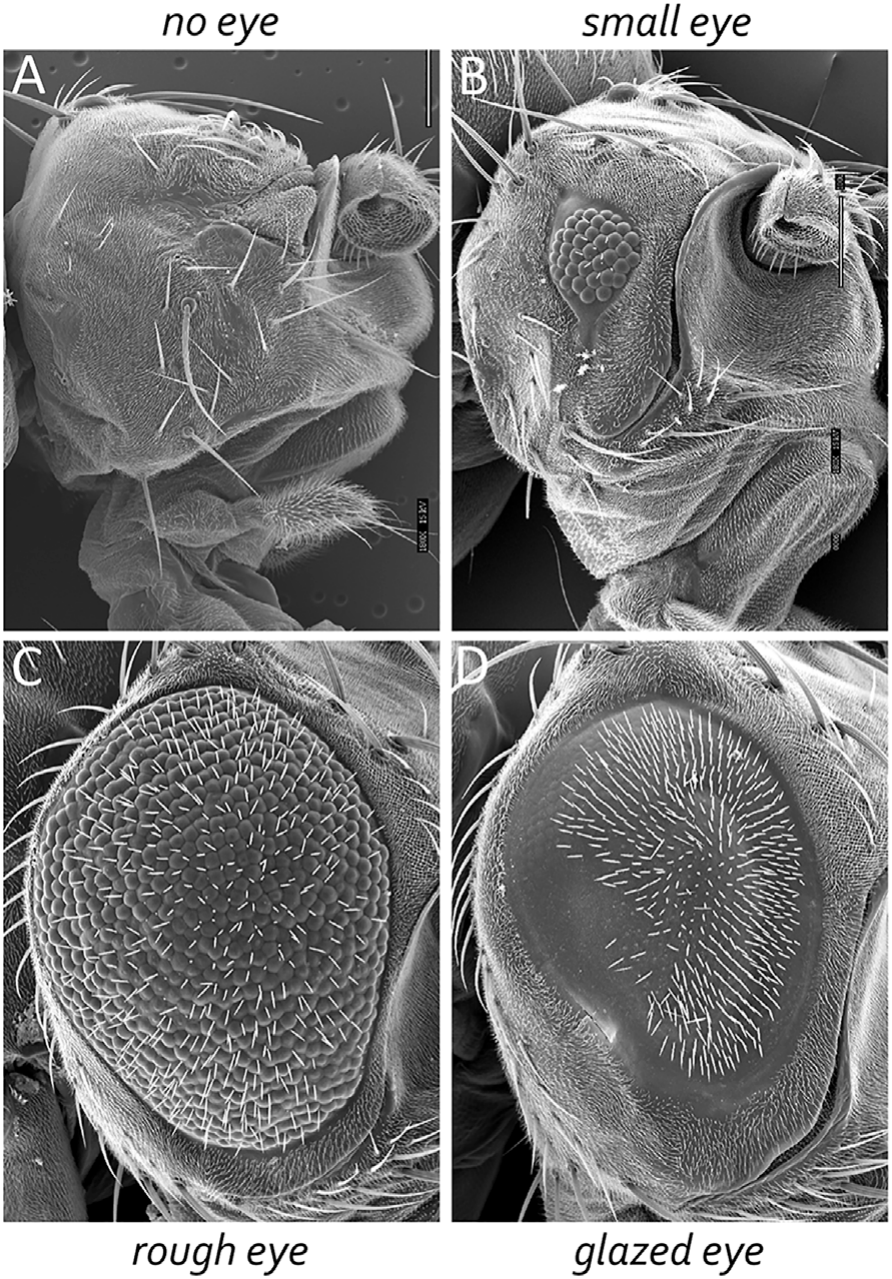
The *Drosophila* compound eye is a model system for identifying genes involved in development. Mutations that affect tissue specification, growth and proliferation, pattern formation, and cell fate specification can be identified by alterations in the crystalline-like nature of the adult compound eye. These mutant phenotypes can manifest themselves as **(A)** the absence of eyes, **(B)** small eyes, **(C)** large-roughened eyes, and **(D)** large-glazed looking eyes.

**FIGURE 7:**
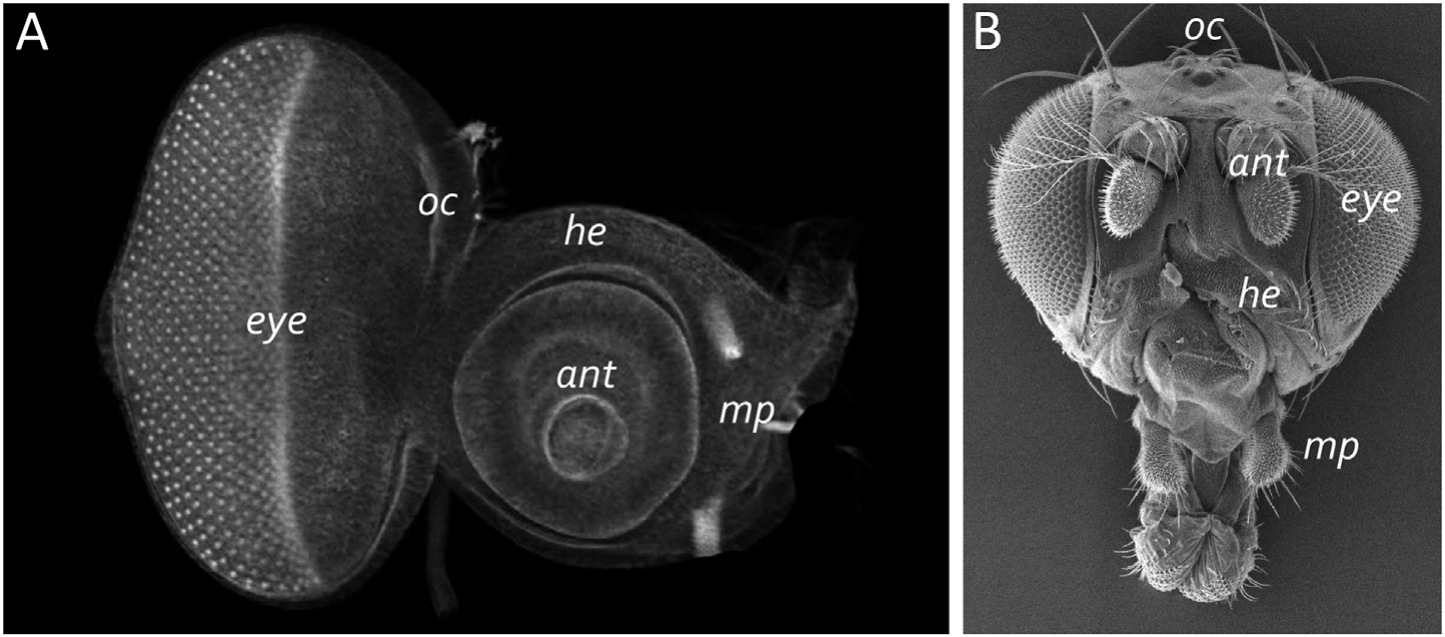
The eye-antennal disc gives rise to the adult head. **(A)** A light microscope image of a third larval instar eye-antennal disc. The disc is divided into several different neighborhoods that each give rise to a unique structure on the adult head. Each larva has two eye-antennal discs that are stitched together during pupal development. **(B)** A scanning electron micrograph of that adult head. The adult structures that are derived from the disc are labeled. oc, ocelli; ant, antenna; eye, compound eye; he, head epidermis; mp, maxillary palp.

The adult eye is derived from a sac-like structure called the eye-antennal imaginal disc (Krafka, 1924; Chen, 1929; Pilkington, 1942). In addition to the compound eye, it also gives rise to nearly all adult head structures including three simple eyes called ocelli, antennae, maxillary palps, and surrounding head epidermis (Figure 6) (Sturtevant, 1929; Bodenstein, 1938; Birmingham, 1942; Zalokar, 1943; Vogt, 1946; Schlapfer, 1963; Abaturova and Ginter, 1968; Ouweneel, 1970; Haynie and Bryant, 1986). The only structural feature of the adult head that is not derived from the eye-antennal disc is the proboscis (mouthpart), which arises from the labial and clypeo-labral imaginal discs (Wildermuth and Hadorn, 1965; Gehring and Seippel, 1967; Wildermuth, 1968; Kumar et al., 1979). The eye-antennal disc, like all other imaginal discs, is comprised of three cell layers—A sheet of columnar cells called the disc proper, a layer of squamous cells called the peripodial epithelium, and a strip of cuboidal cells referred to as the margin (Figure 7) (Atkins and Mardon, 2009; Weasner et al., 2020). The disc proper and peripodial epithelium are of the same overall size and shape and lie juxtaposed to each other. However, due to the differences in the size and shape of the two different cell types that comprise these epithelia, it is estimated that by the end of larval development the number of cells within the disc proper outnumbers those of the peripodial epithelium by a ratio of at least 20:1 (McClure and Schubiger, 2005). These two cell layers are joined together along their edges by the cuboidal margin cells (Figure 7). As such, the eye-antennal disc resembles a closed pillowcase. Enclosed within these three cell layers is a small lumen (Auerbach, 1936) through which signaling molecules are thought to be trafficked either by simple diffusion or through two types of subcellular structures called translumenal extensions and cytonemes (Ramirez-Weber and Kornberg, 1999; Cho et al., 2000; Gibson and Schubiger, 2000; Gibson et al., 2002; Roy et al., 2011).

## Discovery of the morphogenetic furrow

**FIGURE 8:**
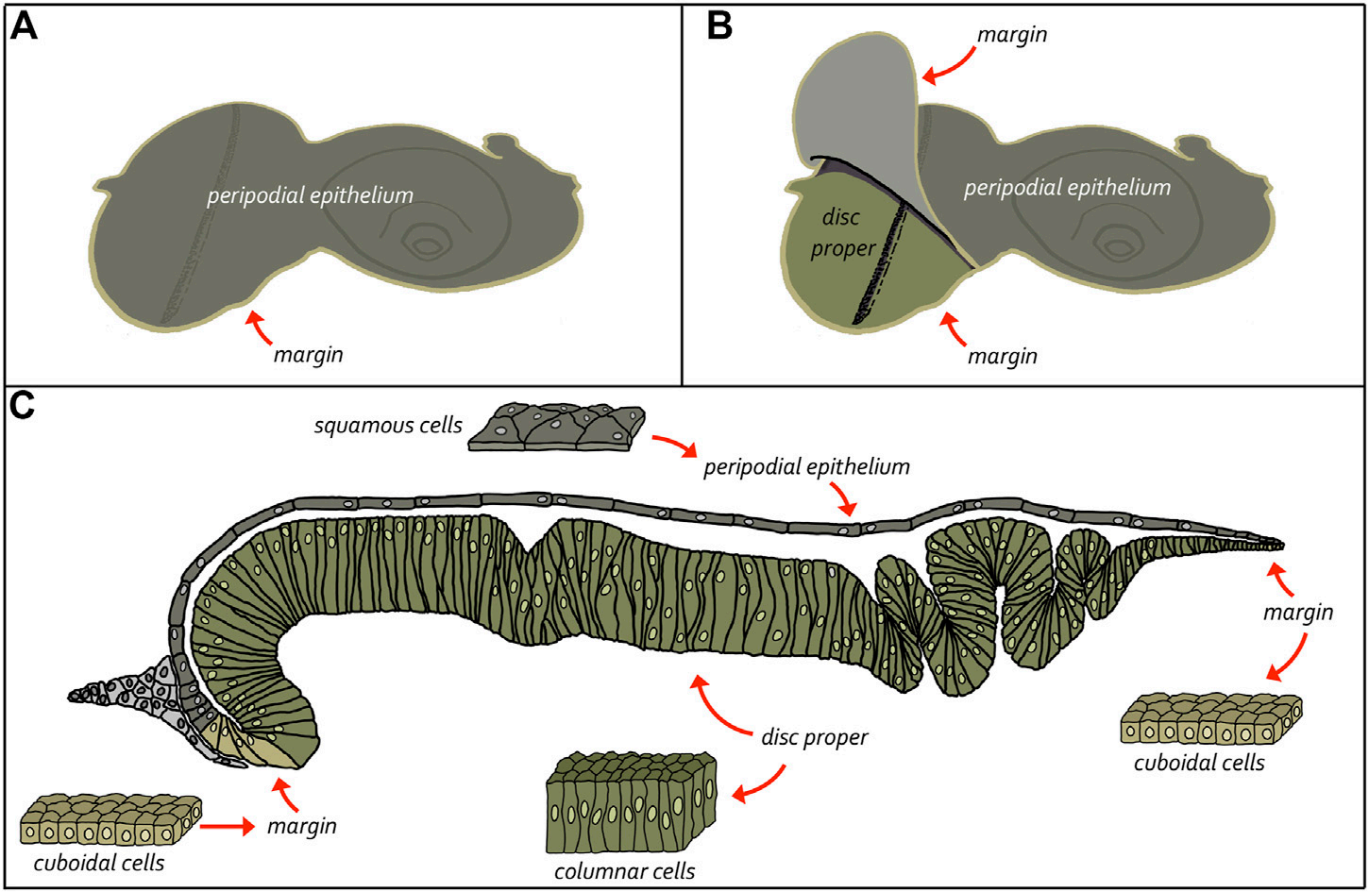
Structure of the eye-antennal disc. The eye-antennal disc is comprised of three different cell types. A layer of columnar cells comprises the disc proper while an overlying layer of squamous cells makes up the peripodial epithelium. These two equally sized tissues are joined together at the edges by a strip of cuboidal cells referred to as the margin. **(A, B)** Schematics showing the relationship between the disc proper and the peripodial epithelium. **(C)** Cross-section view of the eye-antennal disc showing the relative position of all three layers and the enclosed lumen. It also shows the cellular composition of the three cellular layers.

The first recorded description of the eye-antennal disc can be found within August Weisman’s monograph on the development of insects (Weismann, 1864). His camera lucida drawing of the disc includes all known major features including the morphogenetic furrow, which in his drawing appears as a vertical line within the posterior oval domain of the disc. At the time, Weissman proposed that this line (which he probably envisioned as a physical fold in the tissue) demarcates the border between the developing eye and the antennal fields. Early histological methods, applied to the eye-antennal disc, appeared to confirm this prediction as developing ommatidia were only seen in the most posterior regions of the disc (Chen, 1929; Krafka, 1924; Medvedev, 1935; Steinberg, 1943a; b). This view went unchallenged for 112 years until Donald Ready and Seymour Benzer published their landmark paper on the cellular development of the *Drosophila* eye (Ready et al., 1976). They noticed that the “eye/antenna boundary” that Weissman proposed could be found at different physical positions during development. In younger discs the “line” was closer to the posterior edge of the eye field while it would be found nearer to the antenna in older discs. The shifting position of the “line” corresponded to a shift in the number of unit eyes—Younger discs had fewer ommatidia than older discs. From this it was immediately obvious that the “boundary line” was not the eye/antennal border at all but is instead the leading edge of a differentiating wave (Figure 8). They called it the morphogenetic furrow as its movement across the eye field appeared to transform a sea of undifferentiated cells into a periodic array of unit eyes and it appeared as an indentation in the epithelium (Ready et al., 1976).

## Dynamic properties of the morphogenetic furrow

If development is allowed to proceed at 25°C, the third and last larval instar stage begins at roughly 72 h after egg laying (AEL). At this stage the entire eye field is both unpatterned and undifferentiated. But approximately 6 h later at 78 h AEL the morphogenetic furrow leaves the posterior margin and begins its journey across the epithelium (Spratford and Kumar, 2013). Over the course of two and a half days the furrow patterns the retina by organizing thousands of undifferentiated cells into nearly three dozen columns of unit eyes that ultimately will make up the adult compound eye. The developing retina grows by accretion with each new column of ommatidia being added to the anterior face of the last column. The patterning of the eye resembles a growing crystal so much that, in the title of their original paper, Ready and Benzer referred to the compound eye as a “neurocrystalline lattice” (Ready et al., 1976). This level of perfection is achieved through tight regulatory control of pattern formation, cell fate specification, and planar cell polarity.

The differentiating wave was originally described as a furrow, in part, because scanning electron micrograph images of larval eye-antennal discs showed a dorso-ventral groove within the epithelium (Ready et al., 1976). Cross-sections of the disc showed that cells within the furrow were bottle-shaped with very narrow apical domains and enlarged basolateral sides. In comparison, cells on either side of the furrow are tall and columnar. Dramatic changes in cell shape, such are associated with a broad array of patterning events including tissue invagination, cell ingression, and cell extrusion. The transition from columnar to bottle-shaped cells result from the simultaneous dramatic constriction of the apical profile and the migration of nuclei towards the basal surface (Sawyer et al., 2010; Martin and Goldstein, 2014; Heer and Martin, 2017). If a cell making this transition maintains its cell-cell adhesion with its neighbors, then local deformation of the tissue will occur. In the case of the eye disc a stripe of cells along the dorso-ventral axis all constrict their apical profiles and plunge their nuclei in unison while preserving cell-cell adhesion with adjacent cells. This causes a depression in the tissue that we visualize as the morphogenetic furrow.

How and why does the furrow appear to move across the eye primordium? At the cellular level the movement of the furrow across the epithelium is akin to the “wave” done by fans within a sporting arena. At the beginning everyone in the stadium starts out sitting in their seats. For the wave to initiate, fans in one section will stand up while everyone else remains seated. For the wave to then propagate across the arena folks in the standing section all sit in unison while fans in the adjacent section simultaneously all stand up in concert. As this process repeats itself across all sections, it appears as if a wave is sweeping across the stadium. While the wave appears to move across the arena, the fans have, in reality, not moved from one section to another but instead they simply sit and/or stand in place. One can think of the moving furrow similarly. At the start of third larval instar all cells are fully extended with the apical profiles expanded. Then, at 78 h AEL, a stripe of cells at the posterior margin changes their shape in unison thereby creating a dorso-ventral groove at the posterior edge of the disc. A short time later when those cells extend themselves back into their original position, cells within an adjacent, anterior stripe concomitantly make the opposite decision and become bottle shaped. As this process repeats itself nearly three dozen times it appears as if the furrow rolls across the retinal primordium.

**FIGURE 9:**
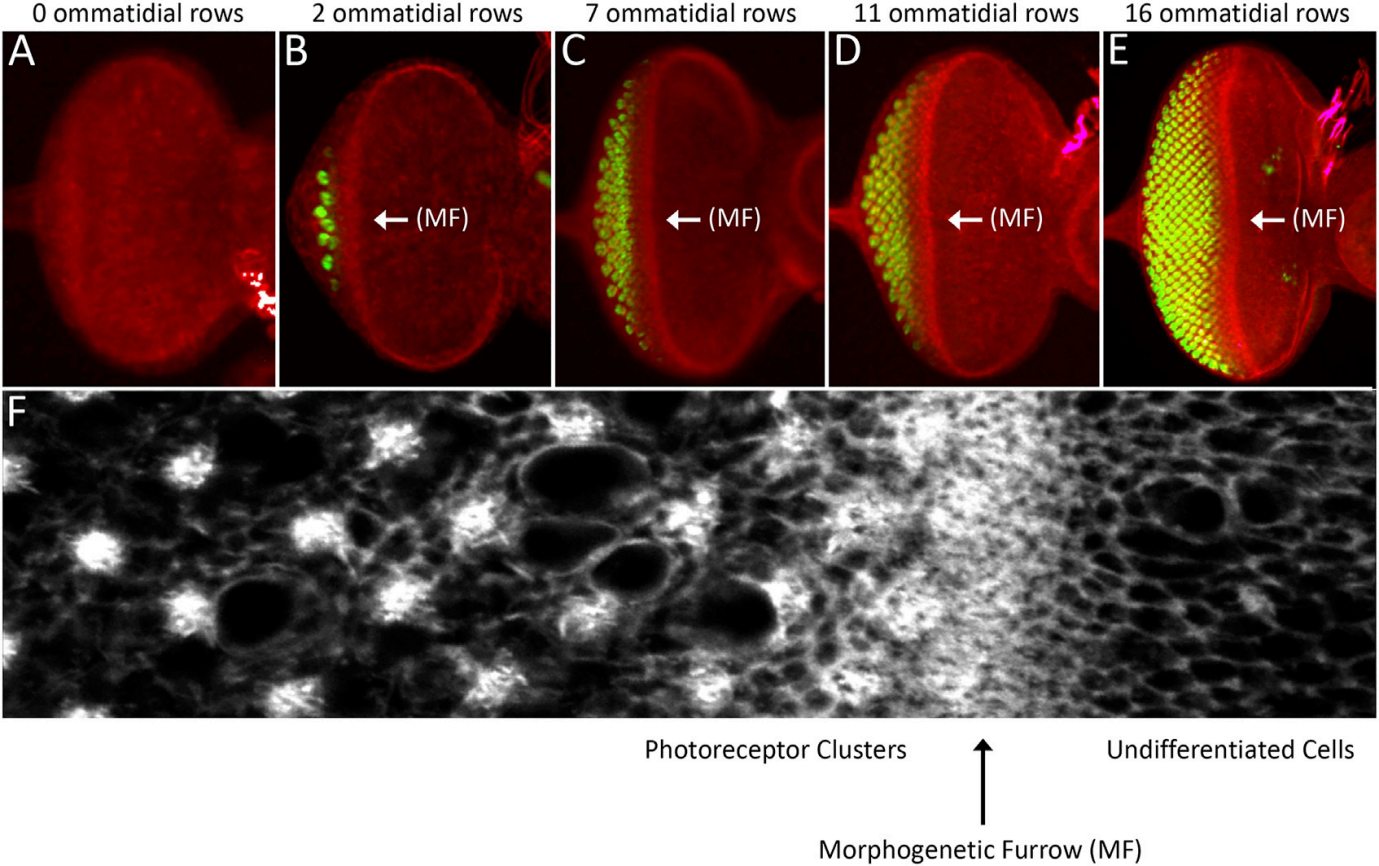
The morphogenetic furrow patterns the eye field during the third larval instar. **(A–E)** Light microscope images of different stage third larval instar eye discs showing the progression of the morphogenetic furrow. As the furrow passes across the epithelium, columns of photoreceptor clusters (ELAV, green) are produced in its wake. **(C)** High magnification view of the area surrounding the morphogenetic furrow. Cells ahead the furrow have large apical profiles and are dividing randomly. As the furrow approaches, cells constrict their apical profiles and enter G1 arrest. As cells exit the furrow groups of periodically spaced cells exist the cell cycle and form the first five photoreceptors of each unit eyes. Cells between these developing ommatidia will eventually undergo one final round of mitosis and give rise to the final three photoreceptor neuron and twelve non-neuronal accessory cells. **(F)** A high magnification image showing a region around the morphogenetic furrow.

The developing compound eye is patterned by the morphogenetic furrow over the course of two and a half days. Several studies have provided differing accounts of how quickly the furrow produces a column of unit eyes as it traverses the eye disc. In the first study, the authors injected third instar larvae with radiolabeled thymidine which would be incorporated into the genomes of cells that were dividing at the time of injection. Cells that took up the radiolabeled thymidine would differentiate into photoreceptors and could be identified later in a retinal section of the adult compound eye. As such, one could determine when a particular column of ommatidia had been born by correlating the position of the labeled column in the adult with the time of radiolabeled thymidine injection within the larva. The authors then compared the position of labeled columns in adults to each other after larvae had been injected at different times during the third larval instar. From this it was proposed that a new column of ommatidia was generated every 120 min, and this timing did not vary across the developing eye (Campos-Ortega and Hofbauer, 1977). From this it appeared as if an intrinsic clock existed within the eye disc to produce columns of unit eyes at regular intervals. Support for this idea came from studies of somite development where a similar degree of periodicity was initially observed for the generation of somites in the frog, *Xenopus laevis* (Cooke, 1975) and then later for other species including snakes, zebrafish, chicken, and mice (Gomez et al., 2008). Johnathan Cook and E.C Zeeman proposed a clock and wavefront model to explain this periodicity (Figure 9). In this model a gradient of positional information (i.e., smooth morphogen gradients) would interact with an autonomously acting internal oscillator (i.e., cycling waves of gene expression) to produce new somites at regular intervals (Cooke and Zeeman, 1976; Saga and Takeda, 2001; Hubaud and Pourquie, 2014). Molecular evidence for the clock component first came when repeating waves of Hairy1 expression were observed within the pre-somitic mesoderm (PSM) during development (Palmeirim et al., 1997; Cooke, 1998). Likewise, support for the wavefront part of the model came from the observation that disruptions to the FGF and Wnt gradients within the PSM disrupt somite formation (Dubrulle et al., 2001; Sawada et al., 2001; Aulehla et al., 2003; Naiche et al., 2011). Similar waves and gradients of gene expression are observed in the developing eye.

A later attempt to document the velocity of the furrow suggested that patterning of the eye field was less uniform than previously thought. The authors first determined that a very brief pulse of Sevenless protein can restore R7 development to a narrow stripe of ommatidia—Sometimes just one or two columns wide. If the authors introduced a pause between two brief pulses, then the velocity of the furrow could be calculated by dividing the intervening time period by the number of ommatidial columns that lacked the R7 cell. From this method the furrow appeared to produce a column of ommatidia every 100 min within posterior regions of the eye which was consistent with the earlier report. However, in anterior half of the eye, the furrow appeared to accelerate and produce a column of unit eyes every 60–70 min (Basler and Hafen, 1989). These two methods indirectly measured the pace at which the retina is patterned. As such, it is not surprising that these studies came up with differing measurements.

A resolution to this issue came when an effort was made to directly measure the rate of patterning across the eye field. To do this, the number of ommatidial columns were directly counted in eye discs from third instar larvae that were carefully timed and dissected at 3-h intervals. From this effort, it appears that patterning is a very dynamic process with the generation time for producing a single ommatidial column ranging from 35 to 150 min. Ommatidial columns at the posterior and anterior edges of the eye field are produced more quickly than those within the center (Spratford and Kumar, 2013). Such differences in velocity suggest that molecular “accelerator and brake pedals” likely exist within the disc to either speed up or slow down the furrow depending upon its position within the disc. At least two brake pedals have been identified—One is a nuclear hormone receptor encoded by the *ultraspiracle* (*usp*) locus and the other is a helix-loop-helix transcription factor encoded by the *extramacrochaetae* (*emc*) gene (Brown et al., 1995; Zelhof et al., 1997; Spratford and Kumar, 2013). The removal of either *usp* or *emc* results in the acceleration of the furrow. In the instance of *emc,* its removal results in a furrow that moves approximately 30% faster than it does during normal development. Emc is able to control the pace of the ommatidial column production by regulating the levels of the activating form of Cubitus interruptus (Ci), the sole transcription factor of the Hh pathway (Spratford and Kumar, 2013). The variable speed at which ommatidial columns are laid down suggests that the mere presence of a repeated pattern in nature does not necessarily guarantee that it is generated with rhythmic periodicity or that it uses an oscillating molecular clock. In short, while somites are generated with a regular periodicity, such regularity does not appear to be a feature of compound eye development. Thus, one can think of the clock and wavefront mechanism as a very specific adaptation of the diffusion-reaction and positional informational paradigms that applies to the production of vertebrate somites.

Molecular accelerators and brakes are required to regulate the pace at which the furrow patterns the eye field because the eye continues to grow while it is being patterned and these two processes need to be synchronized so that 750 ommatidia are generated. If the rate at which the furrow patterns the disc outpaces the rate of cell proliferation, then the resulting adult eye will contain fewer unit eyes than required and will be disorganized. On the other hand, if the furrow moves too slowly then, even though there may be enough cells to make a normal sized eye, fewer than expected numbers of ommatidia will be created by the time larval development comes to an end. This failure to complete eye development on schedule could lead to developmental delays as the fly “waits” for the eye to finish patterning itself. It could also result in a smaller than normal eye if the fly fails to recognize the incomplete state of patterning and proceeds into the pupal stage of development. The furrow must reach the eye/antenna border before the head morphogenesis begins.

### Initiation of the morphogenetic furrow—Evidence for the positional information model

**FIGURE 10:**
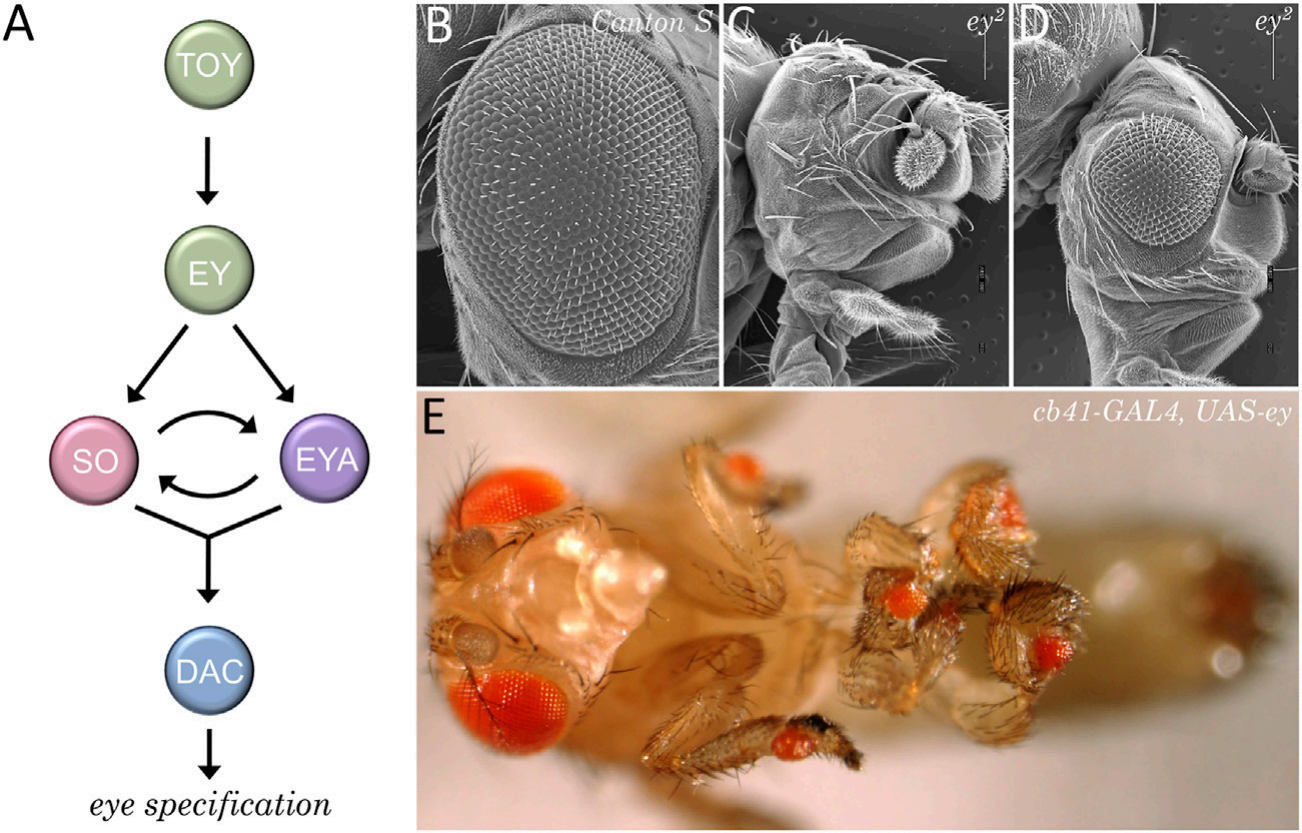
The retinal determination network specifies the fate of the eye. **(A)** The core members of the retinal determination network include the transcription factors Eyeless (Ey), Twin of Eyeless (Toy), Sine Oculis (So), Eyes Absent (Eya), and Dachshund (Dac). **(B–D)** Scanning electron micrographs of wild type **(B)** and eyeless loss-of-function mutants **(C, D)**. Disruptions to the retinal determination network result in either the loss or the severe reduction of the compound eye. **(E)** Forced expression of members of the retinal determination network can induce the transdetermination of non-ocular tissues such as legs, wings, halteres, antennae, and genitalia into eyes.

The positional information model predicts that a source of a secreted morphogen should be present at the posterior edge of the eye field. This is indeed the case in the vertebrate limb bud where Shh is expressed in and emanates from the posterior domain (Riddle et al., 1993; Chang et al., 1994). Around the same time of this discovery there was a lot of interest in understanding how the fly eye was patterned. In decades past, a considerable number of mutants with severely reduced or missing compound eyes had been identified. These mutants were starting to be examined and the underlying genes were being cloned. Several genes such as *eyeless* (*ey*), *eyes absent* (*eya*), *sine oculis* (*so*), and *dachshund* (*dac*) turned out to be core components of the retinal determination network (Kumar, 2010). In addition to the eye being lost in these mutants (Bonini et al., 1993; Cheyette et al., 1994; Hoge, 1915; Mardon et al., 1994; Milani, 1941; Serikaku and O'Tousa, 1994; Sved, 1986) forced expression of these genes in non-retinal tissues is sufficient to induce the formation of ectopic eyes within the antenna, leg, wing, and genital imaginal discs (Halder et al., 1995a; Bonini et al., 1997; Pignoni et al., 1997; Shen and Mardon, 1997). As such, these factors function as selector genes for the eye during the earliest stages of development (Figure 10).

The identification of the retinal determination network had a profound impact on our understanding of how fate of the eye is specified. Furthermore, the subsequent identification of these genes within the eyes of all seeing animals resulted in a paradigm shift in our view of how the eye evolved. The traditional view that the eye arose multiple times (Salvini-Plawen and Mayr, 1977; Land and Fernald, 1992) has been replaced with a new view that the eye originated just once during evolutionary history (Halder et al., 1995b; Gehring, 1996; Callaerts et al., 1997; Gehring and Ikeo, 1999).

**FIGURE 11:**
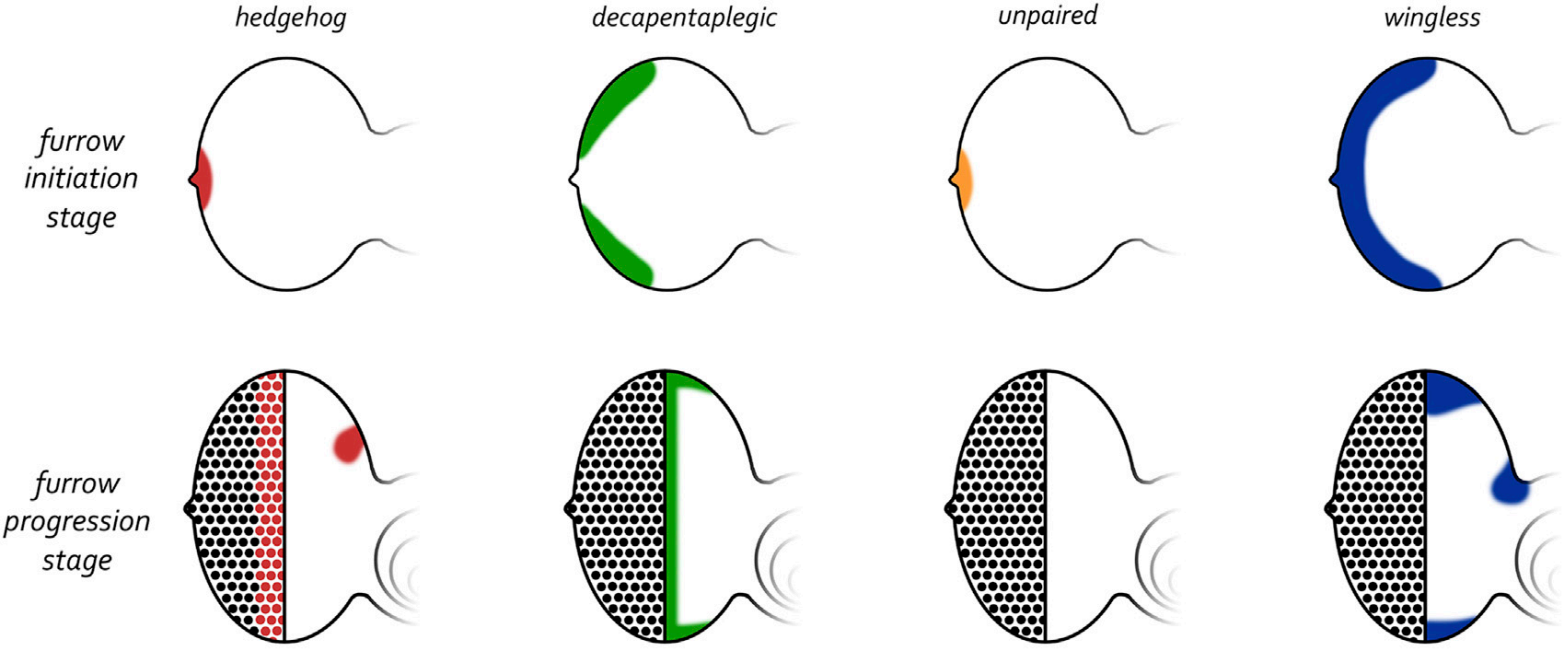
Expression patterns of morphogens that regulate patterning of the eye. (Top row) Early third larval instar just prior to the initiation of the morphogenetic furrow. Prior to the initiation of the furrow, *hedgehog* and *unpaired* are expressed at the firing point while *decapentaplegic* and *wingless* are along the posterior-lateral margins. (Bottom row) Mid third larval instar in which the morphogenetic furrow has progressed half-way across the eye field. As the furrow progresses across the eye field, the expression patterns of all four morphogens are altered dramatically. *Hedgehog* is expressed within the first few columns of photoreceptor clusters, *decapentaplegic* is expressed within cells of the furrow, and *wingless* is expressed ahead of the furrow along the posterior margins. Both *hedgehog* and *wingless* are also expressed within the developing ocellar field.

Prior to the initiation of the morphogenetic furrow, *hh* is expressed at a single point along the posterior edge of the eye primordium (Figure 11) (Ma et al., 1993; Dominguez and Hafen, 1997; Borod and Heberlein, 1998). This is reminiscent of the Shh expression pattern in the limb bud and the asymmetry in *hh* expression within the disc at this stage is entirely consistent with Wolpert’s positional information model. Two lines of evidence proved that the Hh pathway is required for the initiation of retinal patterning. First, a viable mutation within the *hh* locus implicates Hh signaling in the development of the eye. Like several retinal determination mutants, the *hh* ^*bar3*^ allele (renamed *hh* ^*1*^) has very small eyes—Roughly 150 out of a possible 750 ommatidia (Ives, 1950; Mohler, 1988; Renfranz and Benzer, 1989). This defect is caused by a deletion within an eye-specific enhancer element (Pauli et al., 2005; Rogers et al., 2005). It should be noted that in *hh* ^*1*^ mutants the expression of *hh* is severely reduced but not eliminated from the margin and/or photoreceptors neurons. Thus, pattern formation can be initiated and sustained for a short period of time before terminating. Second, if Hh signaling is completely disrupted at the posterior margin then the furrow is prevented from initiating (Dominguez and Hafen, 1997; Borod and Heberlein, 1998; Greenwood and Struhl, 1999; Curtiss and Mlodzik, 2000). Third, ectopic activation of the Hh pathway ahead of the advancing furrow induces undifferentiated cells to initiate ectopic retinal patterning (Chanut and Heberlein, 1995; Heberlein et al., 1995; Ma and Moses, 1995; Pan and Rubin, 1995; Strutt et al., 1995; Wehrli and Tomlinson, 1995; Fu and Baker, 2003). As such, the necessity and sufficiency of Hh signaling in eye development confirms that it can initiate development in a manner predicted by the positional information model.

Grafting experiments using the limb bud had suggested that Shh might function as a long-range morphogen with an activity range of as much as 200 um (approximately 20 cell diameters) (Honig, 1981; Summerbell and Honig, 1982; Riddle et al., 1993; Chang et al., 1994). Similarly, Hh establishes patterning over considerable distances across the *Drosophila* wing and leg imaginal discs (Diaz-Benjumea et al., 1994; Felsenfeld and Kennison, 1995). Initially, it was not clear if Hedgehog proteins exert their influence over such distances by directly acting as long-range gradient morphogens or by functioning as short-range inducers of other downstream signaling pathways. Evidence supporting the latter model came, in part, from studies of the wing and leg imaginal discs. In both tissues, Hh is expressed just within the posterior compartment. Several studies have demonstrated that in the wing and leg, Hh acts locally to activate *dpp* and *wg* within stripes of cells that lie adjacent to the *hh* expressing domain (Basler and Struhl, 1994; Capdevila and Guerrero, 1994; Tabata and Kornberg, 1994; Ingham and Fietz, 1995; Jiang and Struhl, 1995; Li et al., 1995; Pan and Rubin, 1995; Zecca et al., 1995). Their activation is direct as functional binding sites for Ci are present within *dpp* and *wg* disc enhancer elements (Von Ohlen et al., 1997; Muller and Basler, 2000; Parker et al., 2011). The Dpp and Wg signaling molecules in turn, then function as long-range gradient morphogens to pattern the anterior domain of the imaginal discs (Struhl and Basler, 1993; Zecca et al., 1995; 1996). (Nellen et al., 1996) These findings support the clear conclusion that Hh patterns the leg and wing imaginal discs by functioning as a short-range activator of secondary long-range morphogens.

At the start of the third larval instar *dpp* is expressed within domains along the posterior-lateral margins that flank *hh* expression at the firing point (Figure 11). One of the few *dpp* mutants that survive to adulthood contains a deletion of an eye-specific enhancer (*dpp* ^*blk*^). In these mutants, expression of *dpp* along the margins is greatly reduced and as a result, the eyes of these mutants are very small with pattern formation failing to initiate at the dorsal and ventral margins (Borod and Heberlein, 1998; Chanut and Heberlein, 1997a; b). In contrast, if *dpp* expression is forcibly targeted to the anterior margin of the eye field (where it is normally absent), then it is sufficient to initiate a new morphogenetic furrow (Chanut and Heberlein, 1997b; Pignoni and Zipursky, 1997). Together these findings indicate that the Dpp pathway is integral to initiating pattern formation within the retina. In contrast to the wing and leg discs, Hh signaling is unlikely to activate *dpp* transcription within the eye as the onset of *dpp* expression predates that of *hh* during development (Ma et al., 1993; Hazelett et al., 1998; Chang et al., 2001). As we will see in in our discussion of furrow progression, the regulatory relationship between Hh and Dpp that is seen in the leg and wing will return to the retina during furrow progression.

The initiation of the morphogenetic furrow is a very complex regulatory process with several additional signaling pathways contributing to the kickstarting of pattern formation. One key morphogen is Unpaired (Upd), a ligand for the JAK/STAT pathway. At the late second larval instar Upd overlaps with Hh and is present just at the firing point (Figure 11) (Zeidler et al., 1999; Chao et al., 2004; Tsai and Sun, 2004). In contrast to Hh and Dpp, which function to directly promote the initiation of the furrow, Upd and the JAK/STAT pathway appear to control the timing of when the furrow is initiated. High levels of Upd at the firing point correlates with the loss of *wg* transcription, which, until this time, had been expressed along the posterior margin including the firing point (Ma and Moses, 1995; Treisman and Rubin, 1995; Hazelett et al., 1998). As we will see below, early in development the Wg pathway functions at the posterior margin to prevent temporally precocious initiation of the furrow. The JAK/STAT and Dpp pathways relieve this repression at the L2/L3 transition by inhibiting *wg* expression (Dominguez and Hafen, 1997; Ekas et al., 2006; Tsai et al., 2007). By expelling the Wg morphogen from the firing point and posterior margin, the Hh and Dpp cascades are free to initiate the morphogenetic furrow.

Comparing the temporal and spatial expression patterns of *hh*, *upd*, and *dpp* hinted that the initiation of the morphogenetic furrow could be divided into two phases with each chapter being controlled by unique combinations of signaling gradients. The first phase of initiation can be thought of as the primary ignition step. It takes place at the firing point, generates the first column of ommatidia, and is controlled by the Hh and JAK/STAT pathways. Mutations that affect these pathways at the firing point, as expected, often result in a complete block in pattern initiation—Imaginal discs and adult flies lack photoreceptor neurons in these instances (Dominguez and Hafen, 1997; Ekas et al., 2006). In addition to the Hh and JAK/STAT cascades, the EGF Receptor (EGFR) pathway appears to also participate in regulating this phase. Disruption of this pathway using a temperature sensitive allele (*Egfr* ^*tsla*^) identified a critical window where EGFR signaling is required for the initial phase of pattern formation. The furrow fails to initiate when pathway activity is eliminated during this window (Kumar and Moses, 2001). Furthermore, Hh appears to lie downstream of the EGF receptor cascade as its expression is lost when pathway activity is compromised. Furthermore, activation of EGFR pathway activity along the margins is sufficient to initiate new differentiating waves indicating that this pathway is both necessary and sufficient for the initial triggering of retinal patterning (Kumar and Moses, 2001).

The generation of each of the remaining columns of unit eyes can be conceptually thought of as being part of a second phase of pattern initiation. The production of each column involves the progression of the furrow through the middle of the disc (discussed below) *and* the re-initiation of the furrow at the margins. The combination of furrow progression and re-initiation results in a uniform straight wave of differentiation. At the margins, the second phase of patterning relies on the use of the Dpp morphogen, as lowering of Dpp signaling directly or *via* disruption of upstream regulators blocks its re-initiation from the margins (Chanut and Heberlein, 1997a; b; Hazelett et al., 1998; Pignoni et al., 1997). Likewise, blocking either EGFR and/or Notch signaling along the margins prevents the furrow from reinitiating as well (Kumar and Moses, 2001). In all three instances the furrow starts at the firing point and bulges outward—Much like toothpaste being squeezed out of a tube—Instead of appearing as a uniform line across the dorsal-ventral axis.

## Limiting pattern initiation to a single firing point

As we have seen above, prior to the initiation of pattern formation, expression of several morphogen ligands is limited to either the firing point (Hh, Upd) or the posterior margin (Dpp). This restriction is important as ectopic activation of these ligands ahead of the furrow or at any point along the dorsal, ventral, or anterior margins results in the initiation of ectopic differentiating waves (Chanut and Heberlein, 1995; 1997b; Heberlein et al., 1995; Ma and Moses, 1995; Pan and Rubin, 1995; Strutt et al., 1995; Wehrli and Tomlinson, 1995; Pignoni and Zipursky, 1997; Ekas et al., 2006; Tsai et al., 2007). The endogenous and ectopic patterning waves crash into each other within the middle of the eye field resulting in small, drastically disorganized eyes. Thus, the initiation of pattern formation, particularly in the context of an organized, repeated pattern, needs to be restricted to a single point within the epithelium. What is the mechanism(s) that restricts the sources of positional information? Initial insight into this question came from experiments in which temperature sensitive alleles of *wg* were used to inhibit pathway activity within the retina (Ma and Moses, 1995; Treisman and Rubin, 1995). Early in development, *wg* is expressed broadly along the entire posterior lateral margin (Figure 11). But at the end of the second larval instar *wg* expression is “pushed” off the posterior margin and is now restricted to the dorsal-lateral and ventral-lateral margins—These lie just ahead of and abut the *dpp* expression domain (Figure 11). The loss of Wg pathway activity at the start of the third larval instar results in the initiation of ectopic differentiating waves from the dorsal and ventral edges of the eye field. The resulting eyes are small, globe-like, and highly disorganized (Ma and Moses, 1995; Treisman and Rubin, 1995). In contrast, ectopic expression of *wg* within the eye field is sufficient to block both furrow initiation and progression (Treisman and Rubin, 1995; Cadigan et al., 2002). Together, these results indicate that the roles of the Wg pathway is to first block precocious activation of the furrow from the posterior margin (at the L1 and L2 stages) and then later to prevent the emanation of ectopic furrows from the margins (during the L3).

Ectopic differentiating waves are also seeing igniting from the dorsal and ventral margins of eye fields that are mutant for *emc* (Spratford and Kumar, 2013). In *emc* mutants, *wg* expression is lost along the ventral margin suggesting that Emc functions to activate transcription of *wg*. Emc Is a HLH protein and its biochemical function is to bind to and sequester basic helix-loop-helix (bHLH) transcription factors away from their DNA consensus binding sites (Ellis et al., 1990; Garrell and Modolell, 1990; Van Doren et al., 1991). Thus, in this context, if Emc is directly regulating *wg* at the ventral margin, then it does so most likely by removing one or more bHLH transcriptional repressors from the locus. It is alternatively possible that Emc and its binding partner regulates an unknown upstream regulator of *wg*. Interestingly, this genetic relationship does not exist at the dorsal margin—Here *wg* expression remains unchanged suggesting that Emc and the Wg pathway function independently from each other to block differentiating waves from initiating at this margin. It is not clear why the regulatory relationship between Emc and Wg differs at the two margins. But interestingly, in two studies it is reported that the dorsal and ventral compartments of the compound eye are formed at different times and through different molecular mechanisms (Singh et al., 2002; Singh and Choi, 2003; Singh et al., 2004; Won et al., 2015). As such, patterning at the two margins (which border each compartment) could, by extension, be regulated by unique mechanisms.

## Progression of the morphogenetic furrow—Reaction-diffusion makes a temporary comeback

A central tenant of the positional information model and the more recent neighborhood watch model is that cells within a gradient adopt different fates based on the differences in morphogen concentration. This is certainly true of situations like the mammalian limb bud in which distinct types of digits are produced in response to declining amounts of Shh. However, in the developing eye, cells that are close to the original source of Hh (the firing point) as well as those that are located on other side of the disc all make the same decision—All are turned into identically constructed columns of ommatidia. How is this accomplished in the developing fly eye and are there similarities with other patterns in nature that contains repeated elements?

As the furrow begins to traverse the eye disc the expression patterns of both *hh* and *dpp* are completely reconfigured. Expression of *hh* at the firing point is lost and then reinitiated within all newly formed photoreceptor clusters. At the same time, *dpp* expression is extinguished at the margins and is instead activated within the furrow itself (Figure 11) (Heberlein et al., 1993; Ma et al., 1993). The relationship between these two genes now resembles what is seen in the wing in that *hh* and *dpp* expressing cells lie adjacent to each other. Furthermore, genetic studies suggested that Hh functions as a short-range morphogen to activate *dpp* expression within the furrow. Cells within the furrow constrict their apical profiles significantly which, as a result, increases the density of the Patched (Ptc) and Smoothened (Smo) surface receptors—These capture the Hh morphogen (Benlali et al., 2000; Corrigall et al., 2007; Schlichting and Dahmann, 2008). Strong activation of the Hh pathway within cells of the furrow results in the stabilization of the full-length activator form of Ci, which is the sole transducer of Hh signaling (Methot and Basler, 1999; 2001). It is thought to then activate several target genes within the furrow including *dpp* (Dominguez and Hafen, 1997; Greenwood and Struhl, 1999). However, other inputs into *dpp* must exist as it continues to be activated within the furrow even in the complete absence of Ci (Fu and Baker, 2003; Pappu et al., 2003). This is consistent with embryonic and wing development in which Ci appears dispensable for Hh pathway function (Hepker et al., 1999; Gallet et al., 2000). It should be noted that others have come to the opposite conclusion and report that there is an absolute requirement for Ci within the Hh signaling pathway (Methot and Basler, 2001).

Dpp, in turn, then exerts its effects over a longer range and directs a broad swathe of cells ahead of the furrow to prepare to enter a furrow-like state. As cells within the furrow initiate photoreceptor fate specifications, their nuclei rise, their apical profiles expand, they exit the cell cycle, downregulate *dpp* expression, and activate *hh* transcription. Likewise, cells that were once ahead of the furrow now enter G1 arrest, plunge their nuclei basally, constrict their apical profiles, and activate *dpp* expression in response to Hh activity from the newly created column of photoreceptor clusters. This process repeats itself until the furrow reaches the eye/antenna border. These rolling waves of *hh* and *dpp* expression ensure that all regions of the eye field are exposed to the same concentrations of both morphogens at some point during their life history. As such, in the developing eye, the Hh and Dpp pathways generate identical structures (32–34 columns of ommatidia) across the entire eye field. This is markedly different from the mouse limb bud or the leg/wing imaginal discs—In these tissues there is a concentration gradient across the epithelial field such that each cell receives a different dosage of each morphogen and contributes to the development of a unique structure.

When the Hh pathway is activated, the full-length isoform of Ci, which functions as an activator (Ci^A^) is stabilized and prevented from being cleaved into the smaller Ci^R^ isoform. In the wing disc the Hh morphogen gradient translates into opposing gradients of Ci^A^ and Ci^R^ proteins (Parker et al., 2011). Since both activator and repressor forms recognize the same DNA binding site, competition amongst these two forms results in the activation of different target genes along the Hh gradient. Regions with a high Ci^A^/Ci^R^ ratio (near the Hh source) activate *dpp* while cells with a high Ci^R^/Ci^A^ ratio (far from the Hh source) repress *dpp* (Parker et al., 2011). Surprisingly, it has shown that eye development proceeds normally in tissue that completely lacks Ci (Fu and Baker, 2003; Pappu et al., 2003). This finding suggests that, at least in the eye, the Hh pathway may not play a role in directly activating target genes in a concentration dependent manner. Instead, it suggests that the task of the upstream Hh pathway is to eliminate default repression that is caused by the inhibiting form of Ci (Ci^R^). In essence, the role of the Hh pathway will be to prevent the Ci^R^ from repressing target genes such as *dpp*. The conversion of the Hh pathway from a concentration gradient (in the wing) to a binary switch (in the eye) may be essential for generating columns of repeated units as it might allow for the rapid activation and repression of *dpp*—This creates a tight, rolling wave of *dpp* expression.

Turing’s reaction-diffusion model predicted that both activating and repressing signals are required to generate an array of periodically spaced repeated units. In the fly eye, how each unit eye is properly spaced within a column and how each column of unit eyes is appropriately spaced with respect to adjacent columns depends on generating a periodically spaced array of R8 photoreceptors within the eye disc. The R8 cell is the first cell of the unit eye to be specified and it serves as the founding cell for the rest of the ommatidium (Ready et al., 1976; Tomlinson and Ready, 1987; Wolff and Ready, 1991). A key regulatory molecule for R8 specification is the pro-neural transcription factor Atonal (Ato). The R8 photoreceptors fail to form in *ato* loss-of-function mutants and without the founding cell, the remaining photoreceptors, cone, and pigments cells of the ommatidium fail to develop as well (Jarman et al., 1994; Jarman et al., 1995). In contrast, ectopic *ato* expression either due to mis-expression of *ato* itself or through the manipulation of upstream regulators, results in the formation of ectopic R8 cells and a disruption of the ommatidial lattice (Baker et al., 1996; Dokucu et al., 1996; Frankfort et al., 2001; Pepple et al., 2008). Thus, a key step in producing the crystalline like array of unit eyes is to properly activate *ato* expression in a periodically spaced pattern.

**FIGURE 12:**
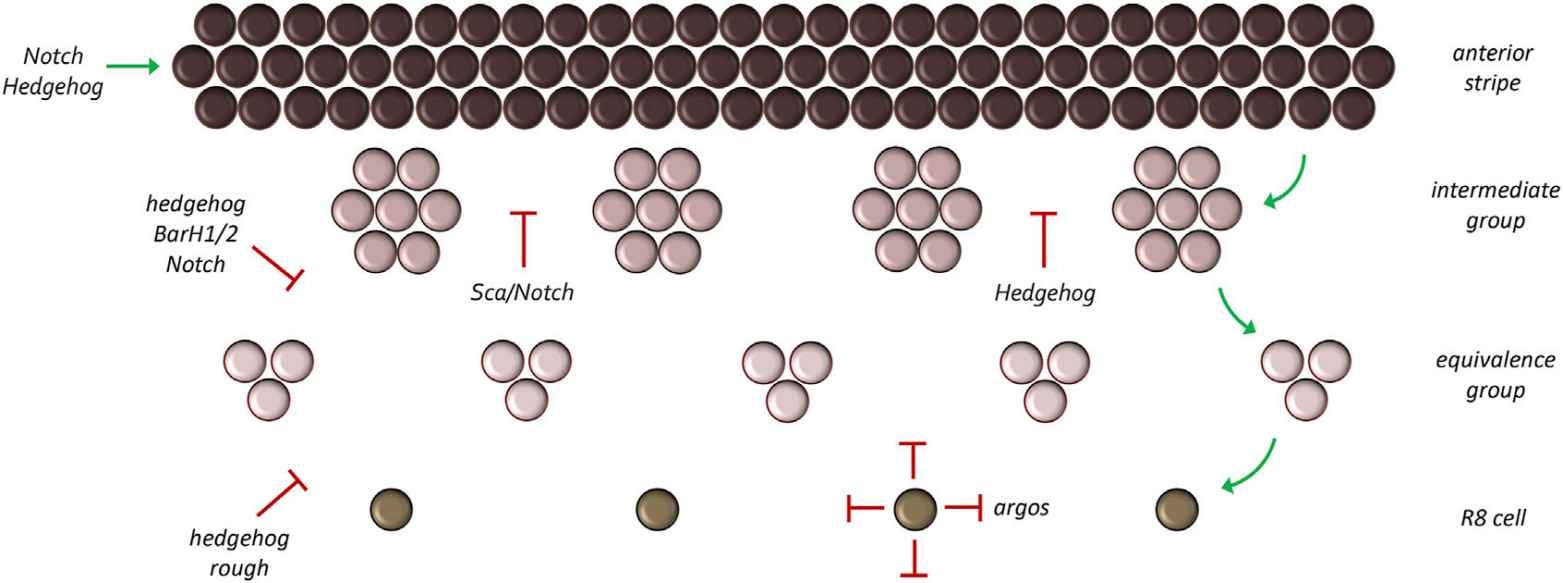
Refinement of the atonal expression pattern. Atonal is first activated in a broad stripe ahead and within the morphogenetic furrow. As development proceeds, this broad pattern is reduced to a column of single cells through a series of refinement steps. During the first step groups of 10–15 cells (called intermediate groups) retain atonal expression. In the second step, atonal expression within the intermediate groups will be eliminated from all but two to three cells—These are now called equivalence groups. In the last step, a single cell within the equivalence group will retain atonal expression. This cell, the R8 photoreceptor, is the founding cell of the ommatidium.

Within the developing eye *ato* expression starts off in a broad pattern which is then progressively reduced to a column of evenly spaced single *ato* positive cells behind the furrow (Figure 12) (Jarman et al., 1994; Jarman et al., 1995; Baker et al., 1996; Dokucu et al., 1996; Frankfort and Mardon, 2002). *ato* is first expressed in a wide dorso-ventral stripe just anterior to and within the morphogenetic furrow. As these cells mature through the furrow and exit on the posterior side, *ato* expression is lost in small periodically spaced cell clusters. What remains are evenly spaced groups of approximately 10–15 Ato positive cells termed intermediate groups. As these cells continue to mature behind the furrow, *ato* expression is extinguished in all but two to three cells per cluster. These cells are considered the R8 equivalence group as each cell has the potential to develop into the R8 neuron. A final refinement takes place so that only a single cell is selected from this equivalence group to become an R8.

Initially, Hh signaling, first emanating from the firing point (during initiation) and later from photoreceptor clusters (during progression), plays a role in activating the broad stripe of *ato* expression (Strutt and Mlodzik, 1996; Dominguez and Hafen, 1997; Borod and Heberlein, 1998). This pattern is controlled by an enhancer element located at the 3′ end of the locus (Sun et al., 1998). As we have seen above, Hh signaling is unlikely to be directly activating target genes such as *ato*. Instead, Hh signaling most likely contributes to the activation of the broad *ato* stripe by eliminating the Ci^R^ isoform. The independence of *ato* from direct Hh signaling is also seen within the Bolwig’s organ, which is used by larvae as a phototactic organ (Suzuki and Saigo, 2000). Direct activation of *ato* in the compound eye comes from the concerted activities of several eye selector genes including Ey, So, and Eya (Tanaka-Matakatsu and Du, 2008; Zhou et al., 2014; Zhou et al., 2016). These are all expressed in broad overlapping stripes ahead of the morphogenetic furrow (Bonini et al., 1993; Cheyette et al., 1994; Quiring et al., 1994; Serikaku and O'Tousa, 1994). In addition to the RD network, the Notch pathway also contributes to the broad stripe of *ato* expression ahead and within the furrow by downregulating levels of the Emc and Hairy (H) transcription factors) which are themselves tasked with repressing *ato* (Brown et al., 1995; Baker and Yu, 1997; Ligoxygakis et al., 1998; Nagel and Preiss, 1999; Baonza and Freeman, 2001). In the eye the Hh and Notch pathways are connected to each other in that Hh signaling activates expression of the Delta ligand which then triggers Notch signaling (Baonza and Freeman, 2001).

As discussed above, the broad band of *ato* expressing cells must be refined into a column of evenly spaced single R8 cells. The process of reducing pro-neural gene expression to single cells is termed lateral inhibition. At each refinement stage, cells that will eventually contribute to future ommatidia (intermediate groups, equivalence groups, single R8 cells) as well as the ommatidia themselves secrete inhibitory morphogens which prevent neighboring cells from becoming photoreceptor neurons. The requirement of inhibitory signals to generate a periodically spaced array of repeated units, be it ommatidia or feather buds, was predicted by Turing and is an essential component of his diffusion-reaction model. Within and behind the morphogenetic furrow, Hh signaling switches from being an activator of *ato* and now, indirectly aids in its repression (Dominguez, 1999). It does so through the default activation of the *BarH1/2* and *rough* (*ro*) genes. Although these transcriptional repressors were originally thought to be activated directly by the Hh signaling pathway (Chanut et al., 2000; Lim and Choi, 2004), the results of Nick Baker and Graeme Mardon suggest that their activation might result, in part, from the elimination of the Ci^R^. Irrespective, both factors are expressed broadly within the furrow (Kimmel et al., 1990; Dokucu et al., 1996; Lim and Choi, 2003) and function to prune the broad band of *ato* expression into single R8 cells with BarH1/2 being responsible for the refinement to intermediate and equivalence groups and Ro for the pruning down to a single R8 cell. Loss of either repressor results in ectopic R8 formation (Heberlein et al., 1991; Dokucu et al., 1996; Chanut et al., 2000; Lim and Choi, 2003; 2004).

The Notch pathway is also redeployed for lateral inhibition and aids the Hh pathway in refining *ato* expression. Loss of Notch signaling during the different refinement stages results in extra R8 photoreceptors and abnormal spacing between ommatidia (Cagan and Ready, 1989b; Baker et al., 1990; Baker and Zitron, 1995). Since the distance between *ato* clusters is too great for Notch-Delta interactions to mediate lateral inhibition, a secreted morphogen with a much longer range is required. One such factor is encoded by the *scabrous* (*sca*) locus which encodes a secreted protein that shares homology with both fibrinogen-related and tenascin extracellular matrix proteins and binds to the Notch receptor (Lee et al., 1996; Powell et al., 2001). As with reductions in Notch signaling, loss-of-function mutations in *sca* also result in extra R8 cells that are too closely spaced to each other (Baker et al., 1990; Mlodzik et al., 1990; Ellis et al., 1994; Baker and Zitron, 1995; Gavish et al., 2016).

The Epidermal Growth Factor Receptor (EGFR) signaling cascade is an important contributor to the spacing of ommatidia but does so later than either the Hh or Notch pathways. EGFR signaling does not appear to play a role in R8 selection as both specification and spacing of this pioneer neuron are completely normal when the receptor is removed (Kumar et al., 1998; Yang and Baker, 2001). R8 specification and spacing are also unaffected if a ligand for the receptor, Spitz (Spi), is likewise removed (Tio and Moses, 1997). Spi contains a single EGF-like repeat and once secreted serves to activate the EGFR signaling cascade (Schweitzer et al., 1995b). Spi is not required for the specification of the R8 cell itself but must be secreted by the R8 for the recruitment of the R2/5 pair of photoreceptors. It is then made within and secreted from these cells to recruit the R3/4 photoreceptor pair. After the second mitotic wave generates a new pool of cells, Spi generated from the last pair of photoreceptors is sequentially used to first recruit the R1/6 and then the R7 neuron. Except for R8, all remaining photoreceptors within the ommatidium require Spi for their development (Freeman, 1994b; Tio et al., 1994; Tio and Moses, 1997). A similar requirement is seen for the EGF Receptor (Freeman, 1996; Kumar et al., 1998; Spencer et al., 1998; Yang and Baker, 2001).

**FIGURE 13:**
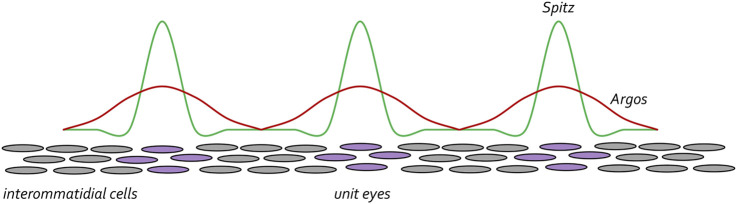
The EGF receptor pathway maintains spacing between developing ommatidial clusters. Once the R8 cell is specified the EGFR pathway is then reiteratively used to specify the remaining photoreceptor neurons (purple circles). Starting with the R8 cell, each photoreceptor secretes the Spitz ligand. This activating morphogen is expressed at high levels but only travels short distances. The photoreceptor cells also secrete the Argos inhibiting morphogen. It is expressed at lower levels than Spitz but travels farther. The differences in levels and distance travelled by the two morphogens ensures that the unit eyes are spaced at periodic intervals. This is reminiscent of the diffusion-reaction model that was proposed by Alan Turing.

To preserve the spacing between unit eyes, mechanisms must be in put in place to limit the range of Spi-EGFR interactions so that cells lying between ommatidial clusters (product of lateral inhibition) do not adopt a photoreceptor fate and join neighboring unit eyes. To do this, the same cells that secrete the activating Spi ligand also secrete a long-range inhibiting ligand called Argos (Aos), which inhibits EGFR signaling *via* two distinct biochemical mechanisms. At one level, Aos inhibits EGFR signaling by competing with Spi for binding to the receptor (Schweitzer et al., 1995a; Sawamoto et al., 1996; Jin et al., 2000). In a second layer of regulation, Aos also binds to Spi and sequesters it away from the receptor (Klein et al., 2004; Alvarado et al., 2006; Klein et al., 2008). Spi is made at higher levels than Aos but diffuses across a much smaller distance. As such, within the cluster of cells that will form the future ommatidium there is sufficient Spi to activate the EGFR pathway. However, since Aos can travel much farther than Spi, Aos-EGFR interactions are dominant in cells the lie between developing unit eyes. Amazingly, built into Turing’s diffusion-reaction model was the understanding that these types of differences in levels and traveling distances of activating and inhibiting ligands are necessary for creating periodically spaced patterns (Figure 13). Loss-of-function mutations in *aos* result in cells that lie between clusters adopting a neuronal fate (Freeman et al., 1992). In contrast, over-expression of *aos* results in too few photoreceptor neurons (Freeman, 1994a; Sawamoto et al., 1994).

As a new column of ommatidia develops, the newly created photoreceptor neurons serve as a fresh source of Hh. As a result, a new band of *ato* expression is activated ahead of the morphogenetic furrow (which has advanced one column) and the entire process begins again. This process will repeat itself until the furrow reaches the eye/antennal border about half-way through the first day of pupal development. The Hh, Dpp, Notch, and EGFR signaling pathways function as the activating and repressing factors within the reaction-diffusion model and their combined activities ensure that an initially uniform field of undifferentiated cells are transformed into a crystalline array consisting of hundreds of identical unit eyes that are organized into several dozen interlocking columns.

## Mechanical cell movements dethrone diffusion-reaction

One of the hallmark features of the *Drosophila* eye is that significant cell movements and/or migrations do not take place during its development (Ready et al., 1976; Campos-Ortega and Hofbauer, 1977; Wolff and Ready, 1991). The lone exception is a set of glial cells that enter the eye field through the optic stalk (Choi and Benzer, 1994; Perez and Steller, 1996; Rangarajan et al., 1999). The cells of the eye field are thought to lie motionless while waves of morphogens pass over them like water rippling over river stones. The small lumen between the disc proper and the overlying peripodial epithelium (Auerbach, 1936) provides a space for morphogens to pass easily over the disc surface without undergoing excessive dilution. From this point of view, cells within the developing eye stand in place and execute their cellular behaviors in response to different combinations and concentrations of the morphogens that we have discussed. When thinking about the movement of the furrow, the optical illusion of the wave moving across a stadium is an apt visual. The static nature of the developing eye was thought to make it an ideal example of Turing’s diffusion-reaction model. In fact, several mathematical models based on reaction-diffusion principles have been specifically proposed to explain how the eye is patterned (Pennington and Lubensky, 2010; Lubensky et al., 2011; Fried et al., 2016; Gavish et al., 2016).

The view that cells within the developing eye lie motionless is based in part on the absence of observable cell migration when discs are extirpated from larvae, cultured in media, and viewed using time-lapse video microscopy (Milner and Haynie, 1979; Milner et al., 1983; Milner et al., 1984; Li and Meinertzhagen, 1995; Tsao et al., 2016; Tsao et al., 2017). However, the culture media used in these assays often did not allow for significant survival times and/or disc growth. In addition, limitations in imaging technology and the absence of advanced imaging software prevented authors of earlier studies from being able to accurately assess cell movements. These technological limitations prevented a rigorous testing of the assumption that little to no cell movements take place within the developing eye field. As a result, researchers have instead relied mainly on snapshots of developing eyes that have been dissected and photographed at successive stages of development and other indirect measures such as the behavior of clonal patches of cells.

A recent study by Richard Carthew’s group describes a breakthrough in developing a long-term *ex vivo* culturing system for the eye-antennal disc. It uses a unique culture media as well as cutting-edge time-lapse microscopy and image analysis to visualize the birth and development of approximately four to five columns of ommatidia over the course of 10–12 h (Gallagher et al., 2022). Their *ex vivo* system is an excellent proxy for studying *in vivo* eye development because all aspects of gene expression, tissue growth, cell fate specification, and planar cell polarity appear to be recapitulated. Surprisingly, and for the first time, the authors noted that there are extensive cellular movements throughout the eye field. Within the morphogenetic furrow itself the authors observed both “fast” and “slow” moving cell clusters—Both of which are moving in the anterior direction. These relative rates refer to the movements of these clusters in relation to each other. The faster moving cells are not yet specified as ommatidia and instead contribute to the leading edge of the morphogenetic furrow. In comparison, the slow migrating cells, by moving slower than the others, appear to emerge from the posterior side of the furrow. A subset of these cells will develop into a column of evenly spaced ommatidia and slow to a standstill. By doing so they fall further and further behind the anterior edge of the furrow. When the developing eye field is viewed in real-time, the faster moving cells appear to race ahead of the new ommatidial column and as such the furrow appears to move forward. The surprising finding that the crystalline growth of the eye is the product of complex cell movements as opposed to diffusion-reaction of morphogens across a sheet of motionless cells is only possible because new cutting-edge technology such as an *ex vivo* culturing system, time-lapse microscopy, and sophisticated image analysis tools were used to revisit a question that seemed to have been answered decades ago.

As we have discussed throughout this article, the Hh, Dpp, Notch, EGFR, and Wg signaling transduction pathways set up chemical gradients that result in repeating waves of gene expression. As a result, a completely non-patterned and undifferentiated sheet of cells is transformed into a neurocrystalline lattice of hundreds of periodically spaced unit eyes. This transformation closely resembles the process of generating the array of feathers on a bird. Based on past assumptions about the static behavior of cells within the eye field, patterning of the eye has been held up as a textbook example of the diffusion-reaction model as was proposed by Alan Turing exactly 70 years ago. However, the mechanical flow of cells that is described by Gallagher et al., 2022 makes the mechanism by which the eye develops incompatible with the diffusion-reaction model as envisaged by Turing.

But models are just that, models. In fact, the positional information model itself is undergoing a makeover as a recent study has proposed a new model to potentially replace it. In the neighborhood watch model, cells do not interpret their position within a gradient in isolation (as predicted by positional information) but instead understand their position within the gradient by comparing themselves to neighboring cells (Lee et al., 2022). In fact, even the role of Shh as a morphogen, which appeared to give proof to Wolpert’s positional information model, has recently been called into question. Instead of acting as a spatial morphogen, Shh is now proposed to act transiently as a trigger for the specification of all digits of the mouse limb in a concentration independent manner (Zhu et al., 2008; Zhu et al., 2022). Similarly, Hh is seen as functioning as binary switch instead of as a morphogen gradient in the fly eye (Pappu et al., 2003). Live imaging of the eye-antennal disc has forced us to view patterning of the fly eye through a completely new lens. It will be fascinating to determine if the signaling pathways that we have discussed here contribute to the flow of cells that appear to exist within the developing *Drosophila* compound eye.

## Concluding remarks

Studies of the *Drosophila* compound eye have, for over a century, made remarkable contributions to our understanding of developmental biology including the mechanisms that underlie tissue determination, specification, and pattern formation (Weasner and Kumar, 2022). The morphogenetic furrow provides a unique opportunity to understand the molecular mechanisms underlying the generation of arrays of periodically spaced identical units, a pattern that is seen repeatedly in nature. While describing the morphogenetic furrow we have endeavored in this article to describe the models of tissue patterning that have underpinned our conceptual understanding of how the fly eye achieves its final crystal-like arrangement. By discussing the morphogenetic furrow within a historical timeline, we hope that we have impressed upon the reader how new technologies can be used to force the fly eye to give up ever more secrets to its development and thus continue to enrich our own understanding of how patterns in nature are generated. Like the initial studies of the furrow, many of the seminal discoveries using the fly eye were made before the molecular age. As such, it is exciting to wonder if applying more sophisticated tools to other aspects of the fly eye will yield new insights into specification and patterning.
